# Supplementary material for: Reconstructing the in vivo dynamics of hematopoietic stem cells from telomere length distributions
Source: eLife. 2015 Oct 15;4:e08687. doi: 10.7554/eLife.08687 (PMC4744200; doi:10.7554/eLife.08687)
Supplement: Supplementary file 1. — (A) Best parameters from fitting the calculated distribution S19 to telomere length distributions of granulocytes from 10 adult persons (see Figure 6—figure supplement 1). Here p denotes the probability that a stem cell proliferation results in two additional stem cells, c is the initial telomere length in kbp and −Δcr/N0 corresponds to the loss of telomere repeats in bp/year. (B) Best parameters from fitting the calculated distribution S19 to telomere length distributions of lymphocytes from 28 adult persons (see Figure 6—figure supplement 2). (C) Best parameters from fitting the calculated distribution S19 to telomere length distributions of bone marrow samples from 28 adult persons (see Figure 6—figure supplement 3). DOI: http://dx.doi.org/10.7554/eLife.08687.017 [file elife-08687-supp1.docx]

**Supplementary File 1A**

Best parameters from fitting the calculated distribution S19 to telomere

length distributions of granulocytes from 10 adult persons (see Figure 6-figure supplement 1). Here $p$ denotes the probability that a stem cell proliferation results in two additional stem cells, $c$ is the initial telomere length in kbp and $-\Delta cr/N_{0}$ corresponds to the loss of telomere repeats in bp/year.

**Supplementary File 1B**

Best parameters from fitting the calculated distribution S19 to telomere length distributions of lymphocytes from 28 adult persons (see Figure 6-figure supplement 2).

**Supplementary File 1C**

Best parameters from fitting the calculated distribution S19 to telomere

length distributions of bone marrow samples from 28 adult persons (see Figure 6-figure supplement 3).
